# Double Cystic Duct in a Septated Gallbladder

**DOI:** 10.1177/2324709615579105

**Published:** 2015-04-09

**Authors:** Wael Otaibi, Giang Quach, Brian Burke

**Affiliations:** 1 St Charles Hospital, Oregon, OH, USA; 2 Oakwood Southshore Medical Center, Trenton, MI, USA; 3 St Elizabeth Medical Center, New York, NY, USA

**Keywords:** double cystic duct, accessory cystic duct, laparoscopic cholecystectomy

## Abstract

Double cystic duct in a single gallbladder is one of the least common variances encountered in the biliary system. This article presents a 54-year-old man who had a septated gallbladder with 2 separate cystic ducts. With intraoperative cholangiogram, he had successful laparoscopic cholecystectomy without any ductal injuries or complications.

## Introduction

Abnormal anatomy of the biliary tree is often encountered during laparoscopic cholecystectomy (LC). These variances predispose patients to higher risks of ductal injury, postoperative complications, and need for convert laparotomy. We present a case of double cystic duct in a septated gallbladder—one of the rarest forms of biliary abnormalities—which was successfully performed laparoscopically.

## Case Report

**Figure 1.**
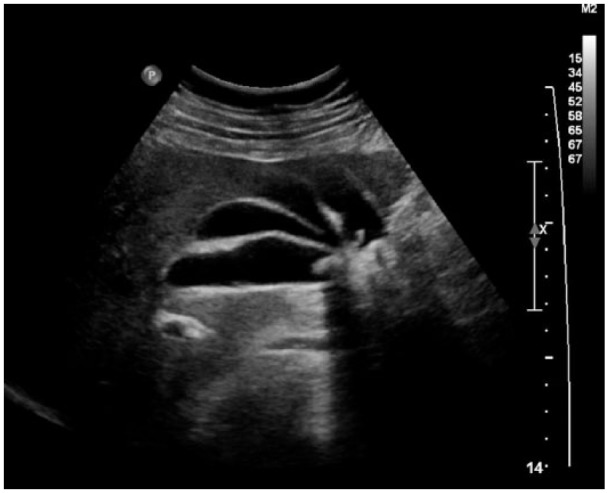
Ultrasound: multiple junctional folds, cholelithiasis. Gallbladder is borderline enlarged.

A 54-year-old male presented with abdominal pain, starting in the epigastrium in the middle of the night with nausea and one episode of vomiting. Physical exam revealed tenderness in the right upper and lower quadrants. Laboratory tests included complete blood count, basic metabolic panel, and liver enzymes. All tests were within normal limits except for slightly elevated alkaline phosphatase, amylase, and lipase. Computed tomography scan showed unusual configuration of the gallbladder, suggestive of septated gallbladder with evidence of cholelithiasis. Radiologist could not exclude a duplication but there was no hint of duplicated cystic duct on computed tomography. Abdominal ultrasound showed cholelithiasis with the gallbladder being borderline enlarged (Figure 1).

**Figure 2.**
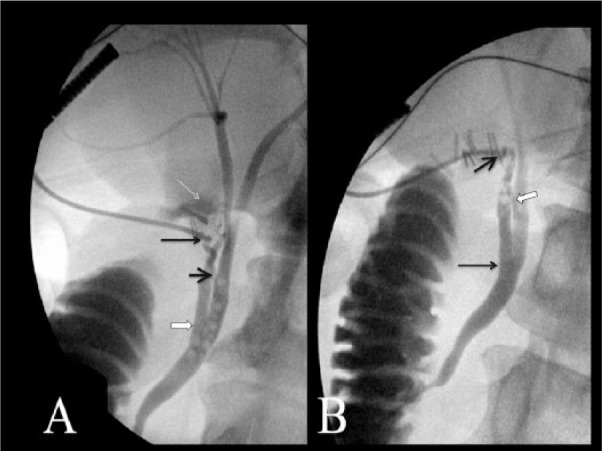
Intraoperative cholangiogram. (A) Initial intraoperative cholangiogram image demonstrates cannulation contrast opacification of a smaller caliber cystic duct (thin long black arrow), which drains into the more superior aspect of the common hepatic duct (short thick black arrow). There is retrograde opacification of the common bile duct and a second larger caliber cystic duct that drains more distal in the common hepatic duct (white arrow with black outline). There is also retrograde filling of portion of the gallbladder from the proximal aspect of the larger caliber cystic duct (long thin white arrow). Multiple filling defects in the common hepatic and common bile ducts are air bubbles. (B) Additional intraoperative cholangiogram after clip placement at the second cystic duct. The second larger caliber cystic duct is cannulated (short thick black arrow) and drains into the common hepatic duct inferiorly (long thin black arrow). There is retrograde opacification of the smaller caliber cystic drain, which drains more superiorly (white arrow with black outline).

The patient was scheduled for LC with intraoperative cholangiogram the next day. After taking down the peritoneal reflection off the triangle of Calot, we identified 3 separate structures. The most lateral structure, connected directly to the gallbladder and appearing to be the cystic duct, was dissected and isolated circumferentially. Cholangiogram was performed using iodinated contrast under fluoroscopic guidance (Figure 2A). We noticed flow into the common bile duct (CBD) and duodenum, and retrograde flow into the common hepatic and intrahepatic radicals. The second structure was found to be connected to the CBD distally and appeared to run parallel to the CBD and end abruptly. On further skeletonizing this structure, it appeared to be connected directly to the gallbladder. A second cholangiogram was performed and found flow through this structure to CBD, duodenum, common hepatic and intrahepatic radicals (Figure 2B). Two separate cystic arteries were identified and clipped. The gallbladder was taken off the fossa and opened for further examination. The configuration of the gallbladder was that of a single gallbladder. Opening it revealed a longitudinal septation with a duct on each side. Both these ducts were shown to open separately into the CBD. The pathologist favored a single gallbladder that was septated. The procedure was completed laparoscopically. Given concern for duct injury due to aberrant anatomy, postoperative magnetic resonance cholangiopancreatography was done, which showed no CBD obstruction or ductal injury. The patient was discharged on postoperative day 2 without any complications.

## Discussion

Although variation in the biliary system is not uncommon, a double cystic duct is quite rare. It is associated with a double gallbladder 80% of the time.^1^ The Harlaftis classification divides these variants into 2 groups.^2^ Type 1 has a single cystic duct and a septum that divides a gallbladder into two. Type 1 can further be divided into 3 subgroups: septated, V shaped, and Y shaped. Type 2 describes accessory gallbladders each with individual cystic ducts.

In 2010, Causey et al described a case of septated type 1 with 2 cystic ducts that are attached to each other, and they postulated that the septation extended all the way down to the level of the CBD.^3^ Our case presents a septated gallbladder but with 2 separate cystic ducts both coming directly from the gallbladder and draining into the common hepatic duct. This is similar to what was found in a cadaver by Aristotle and Jebakani.^4^

Because of the aberrant anatomy, double cystic duct cases predispose patients to higher risks of complications and converting laparotomy. LP has been done successfully with very few instances.^5^ By utilizing intraoperative cholangiogram, we were able to ascertain that the accessory duct was indeed a cystic duct and not an aberrant right hepatic duct. We successfully performed LC without the need to convert to laparotomy.

If preoperative imaging is suggestive of septation or duplication, it behooves the surgeon to fully dissect the triangle of Calot and isolate each structure separately as far proximally toward the CBD as safely possible, and to also free the distal third of the gallbladder from the fossa to obtain a good view of safety as described by Professor Strasberg.^6^ This would put into view any aberrant ductal structure. Cholangiogram should be mandated in these cases and a cholecystogram may actually be more helpful in delineating multiple ducts. As a last resort, conversion to open cholecystectomy may be warranted if confusion or uncertainty regarding the ductal structure persists, to avoid injury to CBD.
